# Supplementary material for: Retracing Schwann Cell Developmental Transitions in Embryonic Dissociated DRG/Schwann Cell Cocultures in Mice
Source: Front Cell Neurosci. 2021 May 20;15:590537. doi: 10.3389/fncel.2021.590537 (PMC8173108; doi:10.3389/fncel.2021.590537)
Supplement: Supplementary file 2 [file Data_Sheet_1.DOCX]

**Supplementary Figures**

**Figure S1: Ki67 ICC DIV1 – DIV7**


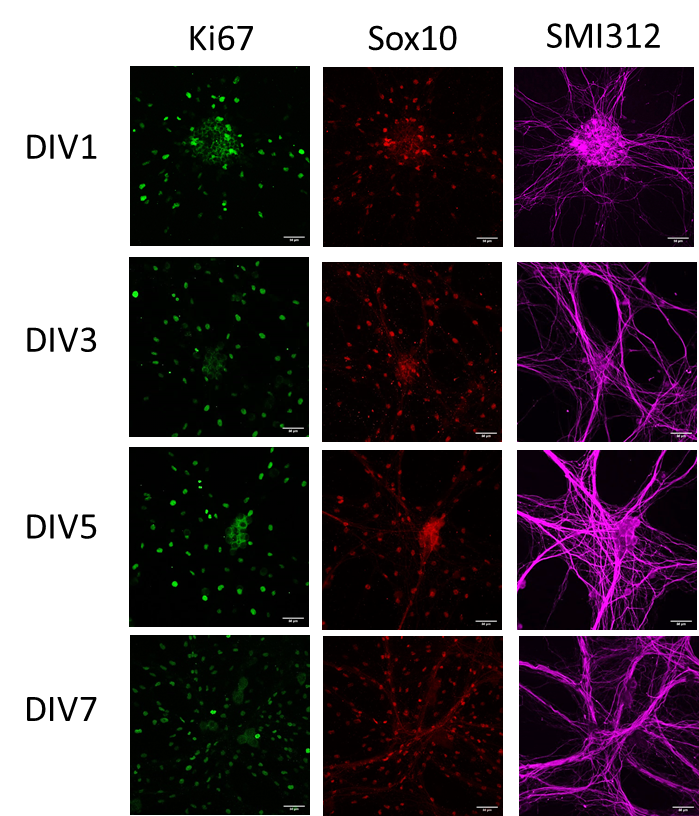


Sox10+ SC are also Ki67+ across all DIVs suggesting that SC continue to proliferate even at DIV7.

**Figure S2: Tfap2α ICC DIV7 vs Prolif. vs Diff**


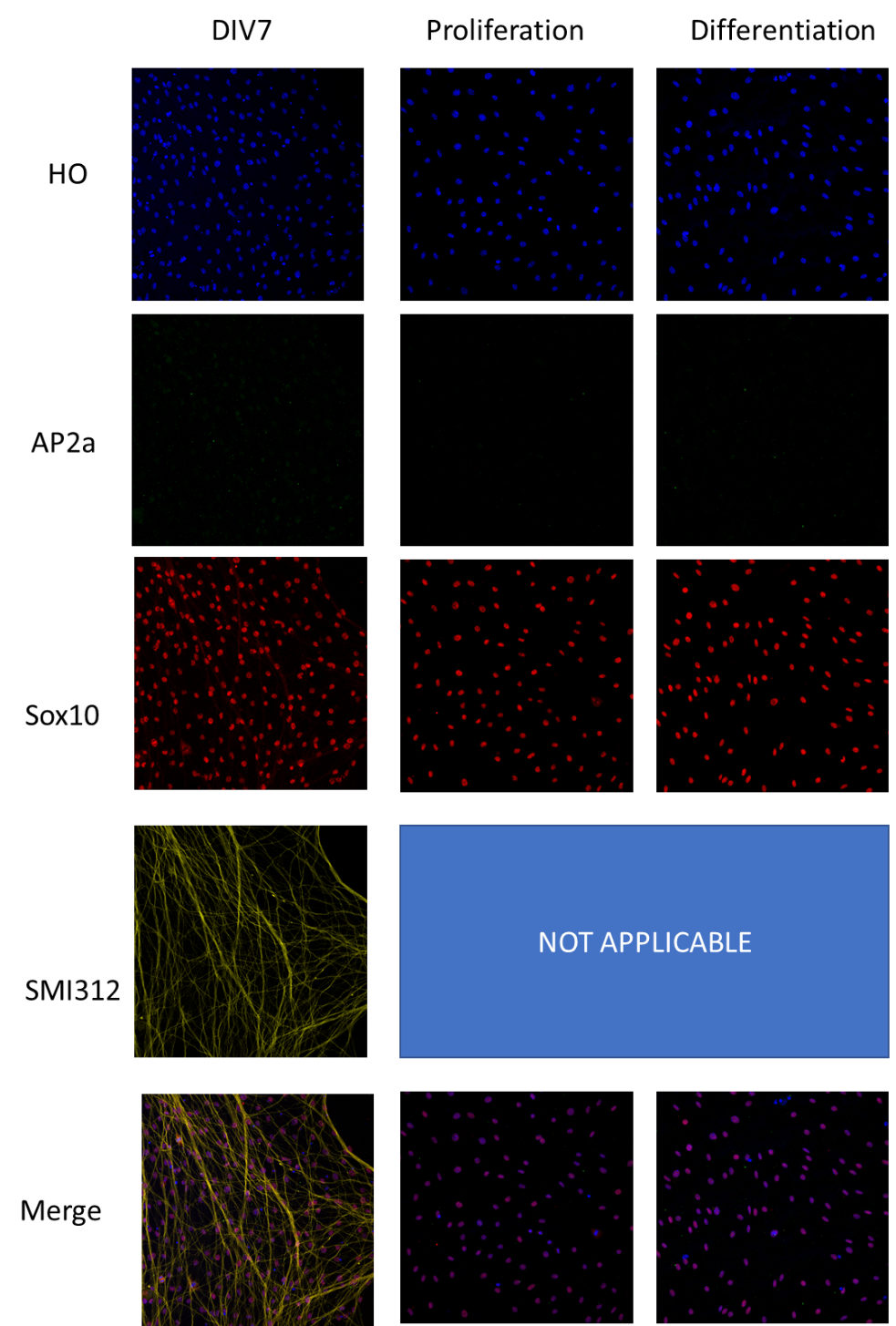


Tfap2α immunoreactivity not detected in any of the three conditions suggesting that cells at DIV7 and in monocultures are not SCP.
